# Endometrial adenocarcinoma presenting as a suprasellar mass: lessons to be learned

**DOI:** 10.3332/ecancer.2020.1083

**Published:** 2020-07-31

**Authors:** Evgenia Granina, Julia Fehniger, Douglas Kondziolka, Joshua Silverman, Andrea Downey, Dimitris Placantonakis, Franco Muggia

**Affiliations:** 1 Department of Internal Medicine, NYU Langone Health, New York, NY 10016, USA; 2 Department of Gynecologic Oncology, NYU Langone Health, New York, NY 10016, USA; 3 Department of Neurosurgery, NYU Langone Health, New York, NY 10016, USA; 4 Department of Radiation Oncology, NYU Langone Health, New York, NY 10016, USA; 5 Department of Pathology, NYU Langone Health, New York, NY 10016, USA; 6 Department of Neurosurgery, NYU Langone Health, New York, NY 10016, USA; 7 Department of Medical Oncology, NYU Langone Health, New York, NY 10016, USA

**Keywords:** stage IA cancer, panhypopituitarism, adenocarcinoma, radiosurgery, HER2/neu, metastases

## Abstract

A 66-year-old woman with a history of stage IA mixed endometrioid and serous endometrial cancer presented to our centre with 2 weeks of worsening headaches nearly 4 years after her initial surgery. At admission, she manifested bitemporal hemianopsia, difficulty walking and clinical and laboratory findings of panhypopituitarism, including diabetes insipidus. Magnetic resonance imaging of the brain revealed a 2.7 cm sellar/suprasellar mass compressing the optic chiasm and infiltrating the pituitary stalk. Computerised tomography documented mediastinal, lung, adrenal and liver involvement, including a 2.5 cm palpable left supraclavicular node that on excisional biopsy demonstrated metastatic endometrial adenocarcinoma. Due to the advanced stage of her cancer as well as the presence of multiple metastases, including lung and hepatic metastases causing post-obstructive pneumonia and coagulopathy, the sellar/suprasellar mass was treated with fractionated radiosurgery rather than surgical excision.

## Introduction

The occurrence of metastatic carcinoma to the sella turcica and the pituitary gland has been previously reported, including one instance in a patient with a history of endometrial cancer [1]. We report another instance that highlights clinico-pathologic aspects of interest, including substantial improvement following stereotactic radiotherapy and a gratifying initial response to carboplatin-based chemotherapy. This benefit was followed by rapid disease progression and underscores therapeutic issues that are central to the management of this all too common cancer in women: recognising which patients benefit most from adjuvant therapy, and the development of systemic regiments upon resistance to platinum-based chemotherapy.

## Case presentation

**Figure 1.**
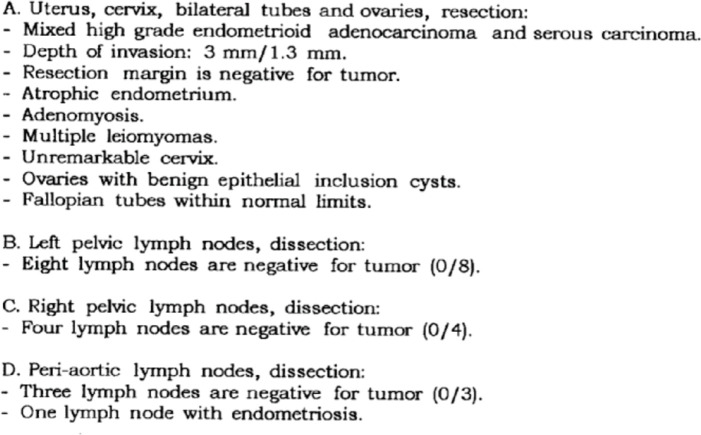
Surgical pathology report (4/23/14).

A 62-year-old non-obese computer scientist, originally born in Azerbaijan, presented to an outside institution in 2014 with postmenopausal bleeding. In September 2014, she underwent robotic-assisted total laparoscopic hysterectomy, bilateral salpingo-oophorectomy and pelvic and para-aortic lymph node dissection. Surgical pathology was consistent with a stage IA (23% myometrial invasion), grade 3, mixed endometrioid and serous endometrial adenocarcinoma (Figure 1). No adjuvant treatment was recommended and she underwent twice yearly routine surveillance physical exams by her gynaecologic oncologist.

**Figure 2.**
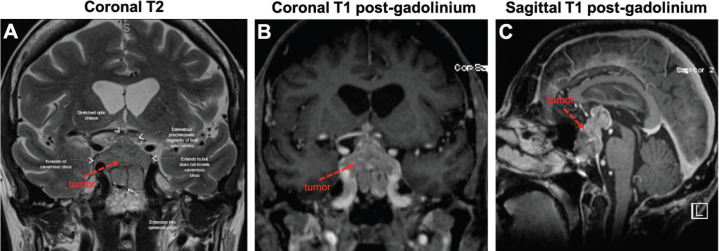
Large sellar/suprasellar mass on MRI. Views of the tumour (red arrows) on coronal T2 (A), coronal T1 with gadolinium (B) and sagittal T1 with gadolinium (C). As pointed out in (A), the tumour invades the right cavernous sinus and extends into the sphenoid sinus. The tumour also compresses the optic chiasm and both optic nerves appear oedematous.

**Figure 3.**
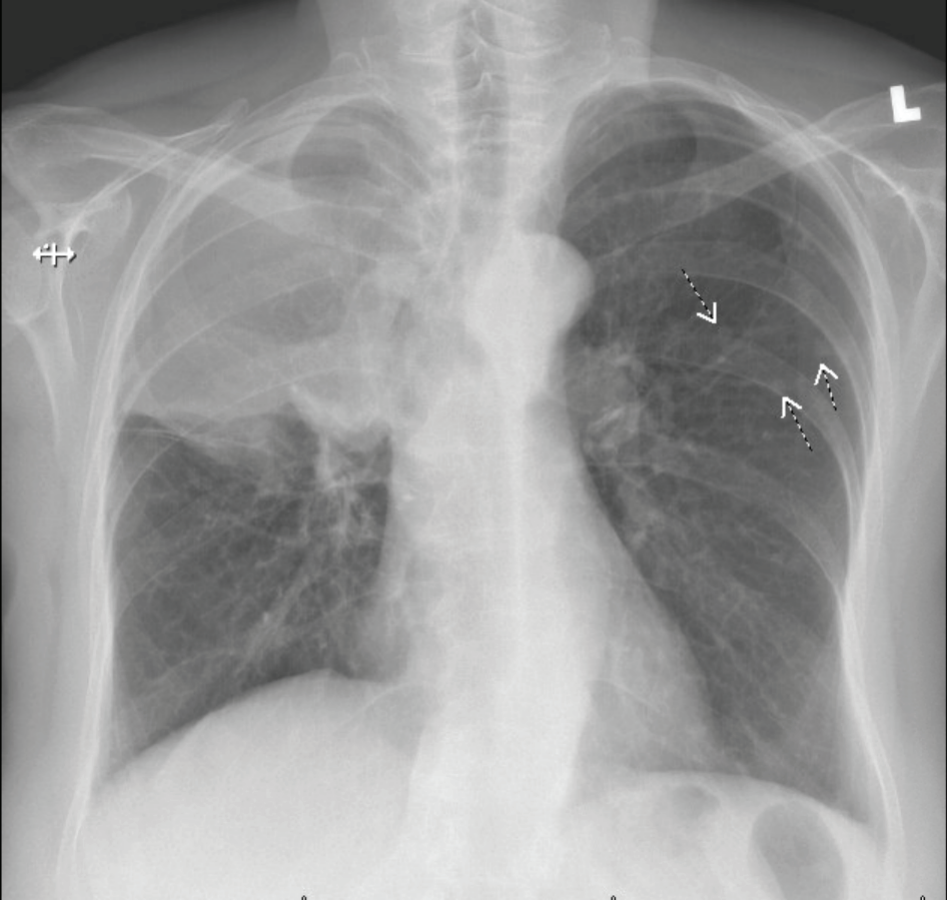
Right hilar mass/opacity with atypical right upper lung zone opacity representing mass or atelectasis. There are multiple smaller nodules in the left chest. Findings suspicious for neoplasia. CT scan of the chest with IV contrast is recommended for further evaluation.

**Figure 4.**
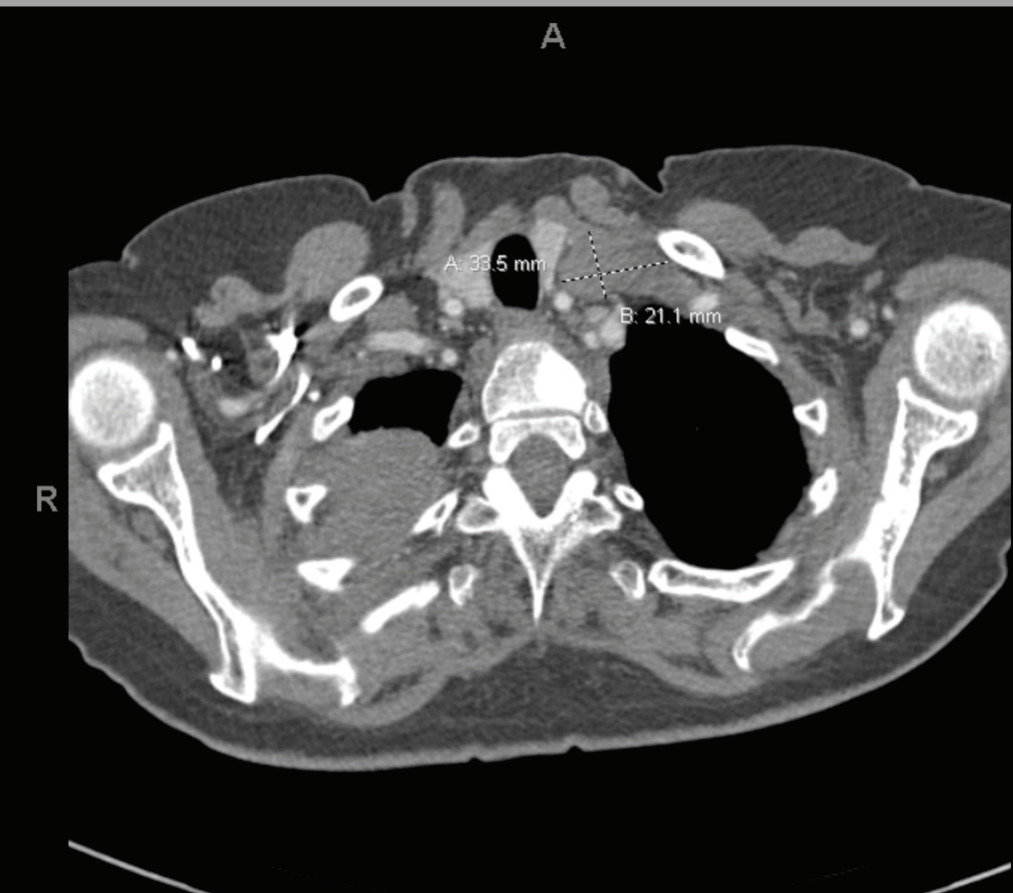
Extensive tumour in the right upper and right middle lobes. Obliteration of the right upper lobe bronchus. There may be some superimposed atelectasis and/or pneumonia. Tumour is contiguous with the right hilum and there is significant adenopathy in the mediastinum and left supraclavicular region. Numerous subcentimetre pulmonary nodules bilaterally, compatible with metastatic disease. T1 sclerotic lesion, worrisome for metastatic disease.

**Figure 5.**
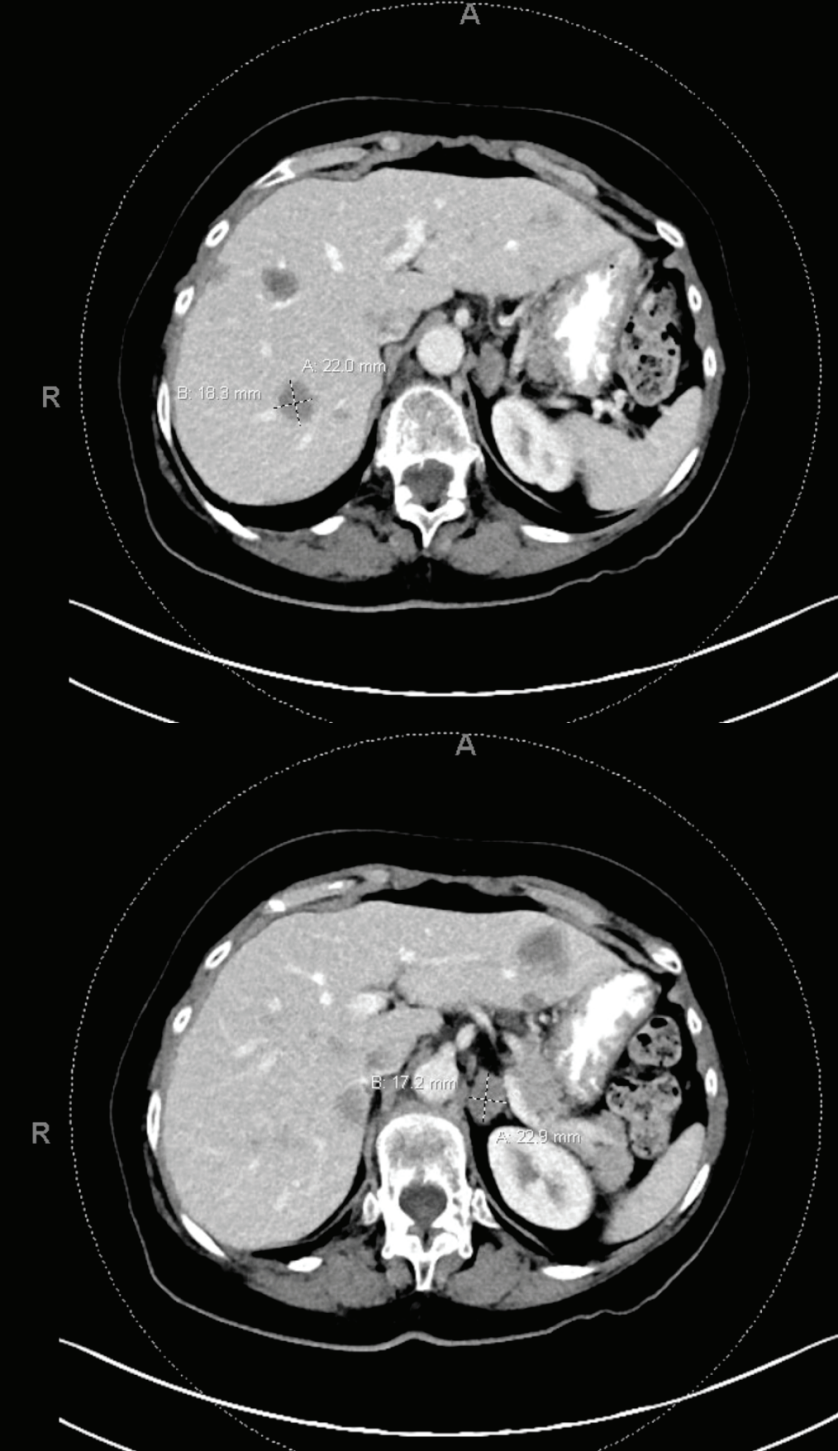
Multiple bilobar hepatic metastases measuring up to 3.2 cm. Bilateral adrenal metastases.

In May 2018, she manifested increasingly severe headaches later associated with dizziness, fatigue and weight loss. In August 2018, she was admitted to our centre with findings of right lid ptosis, right ophthalmoplegia, bitemporal visual field deficits and gait unsteadiness. Endocrine testing revealed low TSH (<0.02), fT4 (<0.5), LH (<0.2) and cortisol (2.2), as well as hypernatremia, suggesting panhypopituitarism and diabetes insipidus. The endocrine deficiencies prompted initiation of replacement therapy. Further inpatient work up included a brain MRI, showing a sellar/suprasellar mass with invasion into the sphenoid and cavernous sinuses, and mass effect on the optic chiasm and hypothalamus (Figure 2). Endoscopic transphenoidal resection was initially planned, but pre-operative chest X-ray and CT (Figures 3 and 4) showed extensive tumour in the right upper and right middle lobes with obliteration of the right upper lobe bronchus as well as hilar, mediastinal and left supraclavicular node involvement. CT abdomen pelvis demonstrated multiple bilobar hepatic metastases measuring up to 3.2 cm and bilateral adrenal metastases (Figure 5). The patient’s clinical course was further complicated by post-obstructive pneumonia and coagulopathy. We, therefore, opted to not resect the suprasellar mass. Excisional biopsy of a supraclavicular node showed metastatic adenocarcinoma of Mullerian origin. Immunohistochemistry demonstrated over-expression of PAX8, TP53, P16, ER and Her-2/Neu (3+). We presumed that the sellar/suprasellar mass also represented endometrial adenocarcinoma metastasis and treated it with three fractions of stereotactic radiotherapy (Gamma Knife) at a fairly low total dose (6Gy × 3). The total tumour volume was 16cc. For hypofractionated radiosurgery, a margin dose of 18Gy was used at the 50% isodose line. Fortunately, this led to an impressive tumour regression for the patient. She maintained visual function and oculomotor function improved.

**Figure 6.**
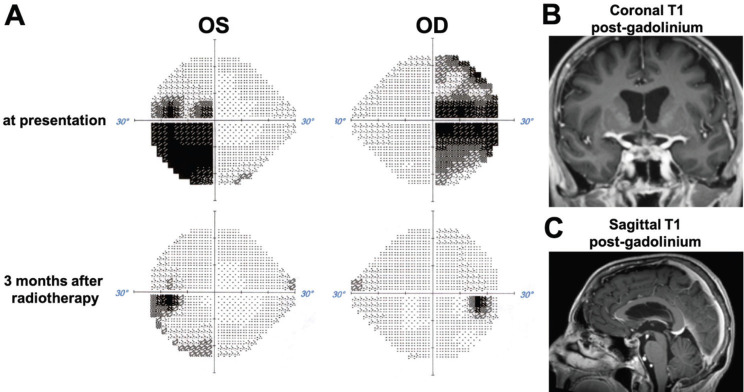
Visual field and radiographic response to radiotherapy. (A) Comparison of Humphreys visual fields for the right (OD) and left (OS) eye at presentation and 3 months after stereotactic radiotherapy. (B) and (C) MRI images obtained 3 months after stereotactic radiotherapy show dramatic regression of the sellar/suprasellar tumour.

Following discharge, the patient began systemic therapy in September 2018 with carboplatin and trastuzumab every 3 weeks. Paclitaxel was initially omitted to avoid impeding the recovery of her improving right ophthalmoplegia. Indeed, while on this regimen, the patient’s right ophthalmoplegia, diplopia and visual field deficits (Figure 6A) totally resolved. Consistent with the clinical improvement, repeat MRI demonstrated a dramatic response of the suprasellar metastasis to the radiotherapy within a few months (Figure 6B and C). We achieved outstanding control of her endocrine deficiencies and water/electrolyte balance throughout her therapy. Repeat CT imaging after five cycles of carboplatin/trastuzumab showed decreased size of metastatic lesions in the lung, liver and adrenal glands. Anticolagulation with apixaban was given after documentation of venous thrombosis in the right femoral vein.

**Figure 7.**
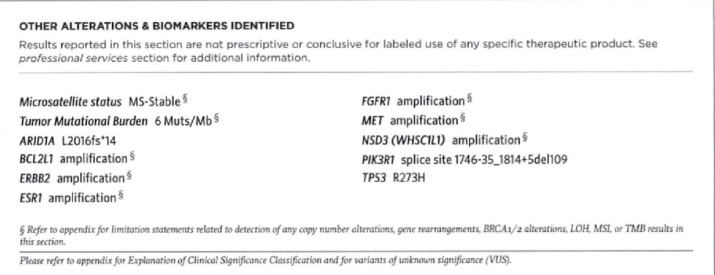
Foundation Medicine molecular analysis. Microsatellite stable. No targetable mutations discovered.

In January 2019, on her sixth cycle of carboplatin (fourth cycle of paclitaxel), the patient experienced an allergic reaction to the carboplatin. Paclitaxel and double antibody treatment with trastuzumab and pertuzumab were initiated. Subsequently, the patient’s CA125 began to rise and she developed hoarseness, cough and left laryngeal nerve paralysis. Imaging subsequently showed a marked regression of the sellar mass and symptoms were attributed to drug side effects. The treatment was initiated with oxaliplatin and pegylated liposomal doxorubicin (Doxil). Unfortunately following four cycles, she manifested progression not only in lung and liver, but also new scattered small brain metastases in the cerebellum, midbrain, left occipital lobe and left frontal lobe. The patient underwent radiosurgery for nine new brain lesions in June, followed by oxaliplatin in July 2019, this time in combination with capecitabine. In August, hoarseness, relentless non-productive cough and eventually haemoptysis prompted withholding anticoagulation. After documenting right major bronchus invasion by tumour, surgical debulking was performed, followed by radiation confined to the bronchus and adjacent mediastinum. Cisplatin 30 mg/m^2^ was also given twice weekly as a radiosensitiser. Surgical tissue was sent for Next Generation Sequencing with Foundation Medicine to check for targetable mutations. The tumour was found to be microsatellite stable and no other targetable mutations than HER2 were found (Figure 7). The initial plan was for the patient to start on Pembrolizumab with Lenvatinib in October 2019 [2]. After receiving one dose of Pembrolizumab, she was found to be too weak to tolerate Lenvatinib. The patient then expressed her desire to stop all therapy. She died at home one week later in November 2019.

## Discussion

Endometrial cancer is the seventh most common malignancy world-wide. In North America, it is the eighth most common cause of cancer death among women [3]. Risk factors include increasing age, unopposed oestrogen exposure, tamoxifen use and obesity. Postmenopausal vaginal bleeding is the usual presenting symptom leading to early detection by sampling the endometrium. Diagnosis and staging include surgical removal and pathologic evaluation of the uterus, cervix, bilateral fallopian tubes and ovaries and regional (e.g., sentinel) lymph nodes. Currently, staging is most often accomplished by minimally invasive robotic surgery that facilitates sentinel node mapping of draining lymph nodes. Outcomes from minimally invasive procedures are similar to those from open, staging procedures and nodal dissection, even in patients with more aggressive histologic subtypes [4, 5].

Atypical sites of metastasis include extra-abdominal lymph nodes, liver, adrenals, brain, bones and soft tissue [6]. Central nervous system (CNS) metastases are generally rare, but in a recent series from University of North Carolina from 2004–2018, 24 cases were identified and 83% of these including ‘high grade’ (i.e., type 2 histologies) [7]. A preceding Mayo Clinic review of 1,632 endometrial cancer included 18 referred for treatment of brain metastases – those with single site of disease had best prognosis [8]. In a Gynaecologic Oncology Group, phase II study investigating the use of pegylated liposomal doxorubicin in 42 patients with recurrent previously treated metastatic endometrial cancer, four patients manifested new brain metastases (two in the cerebellum). Individual case reports describe one case with metastases to cerebellum and one to the pituitary gland [9].

Brain metastases are a significant cause of morbidity and mortality for cancer patients. Without treatment, median survival rates are exceedingly low [10]. Stereotactic radiosurgery (SRS), which involves the administration of targeted high dose radiation to affected areas, has emerged as an effective way to manage systemic disease and improve local control [11]. Prior to the widespread use of SRS, whole brain radiotherapy (WBRT) and surgery were the mainstays of treatment. Both approaches often result in somewhat profound neurotoxicity and a decline in functional status. Although SRS offers the benefit of precision, lower toxicity and shorter treatment course, there has been some debate regarding whether SRS should be offered alone or in combination with WBRT. Our patient initially received SRS alone for a solitary sellar mass with profound results. Subsequently, upon progression of disease, SRS was offered again due to the small volume of lesions despite their significant number (nine lesions in total). WBRT was not offered due to evidence suggesting that there is no difference in overall survival or neurocognitive function when WBRT is given in conjunction with SRS [12]. Due to the number and distribution of brain mets, excisional surgery was not an option, although it should be mentioned that thus far there have been no trials directly comparing surgery to SRS.

In high risk stage I (grade 3 with deep invasion), Stage II, III endometrioid cancers, as well as Stage I–III serous or clear cell histology cancers, randomised clinical trial evidence that the addition of chemotherapy to radiotherapy in the adjuvant setting results in improved outcomes was lacking until PORTEC III. Generally, endometrial cancer carries a favourable prognosis, except in the case of high grade features (such as in our patient). Although the PORTEC III showed an improvement in survival with adjuvant chemoradiation versus radiotherapy alone, patients with Stage III and serous cancers had a higher rate of recurrence than those with Stage I/II cancers or those with other histologic types [13, 14]. It is unclear whether using PORTEC III to guide treatment would have resulted in a different outcome for our patient at the time of her initial diagnosis as this evidence was not yet available. For patients presenting in advanced stages and with evidence of recurrence more than 6 months after adjuvant therapy, treatment with carboplatin and paclitaxel is favoured [15]. After progression from first-line platinum and taxane-based treatment a wide range of options including targeted therapies and best supportive care should be considered [16].

Possible new targeted therapies include HER2 directed therapies, anti-angiogenic drugs, immune check point inhibitors and antibody-drug conjugates. HER2/NEU over-expression is found in 10%–20% of endometrial carcinomas [17] and is associated with late progression and poor survival. Additionally, HER2/NEU overexpression is common in high grade, especially serous tumours [18]. Fleming *et al* [19] did not demonstrate a benefit for single agent trastuzumab in HER2 overexpressed or amplified endometrial cancers. In contrast, Fader *et al* [20] found that the addition of trastuzumab to carboplatin and paclitaxel in patients with metastatic or recurrent uterine serous cancer with HER2/neu overexpression led to improved progression free survival. Interestingly, Teplinsky and Muggia [21] discussed a partial response to single agent lapatinib in persistent or recurrent endometrial cancer. Nevertheless, the routine incorporation of HER2/NEU testing is increasingly being incorporated into the pathologic evaluation of uterine serous cancers to identify patients who may benefit from targeted therapy with trastuzumab. In our patient, however, an initial response to carboplatin and radiation was followed by resistance to other therapies, including those directed against HER2/neu.

## Conclusion

Recurrent endometrial cancer with central nervous system metastasis is a rare presentation of recurrent disease. Of note, stereotactic radiation provided excellent control of the patient’s intraparenchymal cerebral disease. An initial response to platinum-based chemotherapy provided the patient an extended period of well-being. Ultimately however, drug resistance ensued and the patient succumbed to rapid disease progression in pre-existing pulmonary and hepatic metastases. In addition, to the key importance of incorporating new knowledge in tumour biology to our increasingly more precise surgical staging, this case serves as a reminder for clinical trials exploring initial treatments beyond surgery and earlier detection of recurrences during follow-up, including enhanced clinical suspicion of CNS metastases, especially following an initial diagnosis of high grade and serous histologic subtypes regardless of stage.

## Disclosures

None to report.

## Conflicts of interest

There are no conflicts of interest, including financial, non-financial to report.

## Funding statement

The authors received no specific funding for this work.
